# Assessing mesh size and diffusion of alginate bioinks: A crucial factor for successful bioprinting functional pancreatic islets

**DOI:** 10.1016/j.mtbio.2025.102175

**Published:** 2025-08-05

**Authors:** Carolin Hermanns, Rick H.W. de Vries, Timo Rademakers, Adam Stell, Denise F.A. de Bont, Omar Paulino da Silva Filho, Marlon J. Jetten, Carlos D. Mota, Sami G. Mohammed, Vijayaganapathy Vaithilingam, Aart A. van Apeldoorn

**Affiliations:** a Department of Cell Biology – Inspired Tissue Engineering (cBITE), MERLN Institute for Technology Inspired Regenerative Medicine, Maastricht University, Maastricht, the Netherlands; b Department of Complex Tissue Regeneration (CTR), MERLN Institute for Technology Inspired Regenerative Medicine, Maastricht University, Maastricht, the Netherlands; c MERLN Institute for Technology Inspired Regenerative Medicine, Maastricht University, Maastricht, the Netherlands

**Keywords:** Bioprinting, FRAP, Rheology, Type 1 diabetes mellitus

## Abstract

Three-dimensional (3D) bioprinting has been utilised for the encapsulation of pancreatic islets for potentially treating type 1 diabetes. A crucial factor in selecting a cell compatible bioink, that maintains islet functionality, is the mesh size and diffusion capacity of the bioink. In this study, we present a screening strategy for alginate hydrogel formulations in three-dimensional bioprinting, utilizing the fluorescent recovery after photobleaching (FRAP) method and measuring the mesh size of the hydrogels. Subsequently, the 1.5 % alginate formulation that had been selected was used to bioprint the INS1E cell line, primary rat islets, or human islets. It was demonstrated that cell viability and functionality were maintained in all cell sources. This was evidenced by the observation that bioprinted pancreatic islets exhibited a response to physiological glucose levels. The present study indicates that both FRAP and hydrogel mesh size measurements are effective tools for predicting the diffusion of hormones through a hydrogel. These measures should therefore be incorporated into future screenings of hydrogel compositions for the 3D bioprinting of islets.

## Graphical abstract

**Figure:**
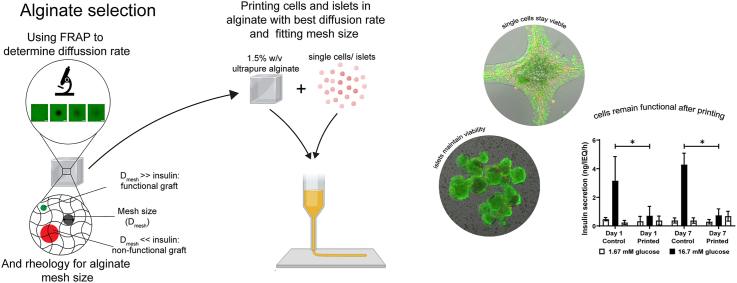

## 1 Introduction

The liver is the implantation site of choice for conventional clinical islet transplantation (CIT), despite being a suboptimal implantation site [1]. Currently, several different micro- and macro-encapsulation techniques are under investigation to improve CIT by realizing extrahepatic transplantation. These techniques either aim to increase islet revascularization or act as an immunoisolation barrier for allogeneic islets. Macro-encapsulation devices carry hundreds to thousands of islets in one single device and have the advantage of easy retrieval after implantation [2,3]. However, it is possible that there is insufficient diffusion of oxygen and nutrients towards the core of the device, which is even more problematic as pancreatic islets have the tendency to aggregate. The aggregated islets form large structures with hypoxic cores, which further deteriorate into necrotic cores [3]. Islet loading density and prevention of severe oxygen competition is therefore the most limiting factor for macro-encapsulation strategies. In contrast, only single or a few islets are encapsulated in microencapsulation strategies, such as hydrogel microcapsules [2,3]. The relative high surface area/volume ratio of microcapsules results in improved diffusion properties compared to macro-encapsulating devices. However, the small size of microcapsules makes them difficult to retrieve after implantation [3]. One leading example of microencapsulation are alginate-based microcapsules [[2], [3], [4]]. Alginate is a natural hydrogel which is used for different biomedical applications such as the delivery of small molecules, proteins and cells due to its biocompatibility, adaptability, low toxicity, low cost, and mild ionic gelation by addition of divalent cations such as Ca^2+^ and Ba^2+^ [5,6]. Hydrogels such as alginate can be tuned to resemble the native cell environment, for instance by controlling the water content and mechanical properties, and are thus a promising solution to create an artificial extracellular matrix surrounding transplanted tissue [7]. Moreover, alginate can act as a physical barrier, preventing immune cells from reaching encapsulated islets and prevent the use of systemic immunosuppression [8].

3D bioprinting combines the advantages of micro- and macro-encapsulation. A 3D printed hydrogel would ideally house large number of islets (multi-layering), shield encapsulated islets from immune cells, facilitate a maximised surface area/volume ratio for optimal diffusion of oxygen and nutrients, while also utilizing a superordinate macrostructure to ease retrieval and provide stability. However, determining the ideal bioink formulation to realize bioprinting of cell-laden constructs remains challenging. The bioink must demonstrate sufficient architectural and mechanical properties to facilitate multi layered printing and handling. It is also imperative that the bioink do not cause cellular damage during printing process, rather maintain cell phenotype, viability and function. [9]. The pore diameter of alginate gels has shown to range between 5 nm and 200 nm which allows diffusion of relatively large molecules through the hydrogel [10]. Therefore, alginate has been used for 3D bioprinting of cartilage, bone and vascular tissue, as previously reviewed [11]. Interestingly, alginate has been used for 3D bioprinting islets.

To date, only a few groups 3D bioprinted pancreatic islets inside a hydrogel and assessed cell viability and function [[12], [13], [14], [15], [16]]. While encapsulated islets maintained their viability in all studies, their function as measured by glucose-stimulated insulin release was compromised. This is most likely due to impaired diffusion of molecules through the hydrogels, delaying or preventing the release of insulin out of the hydrogels. This highlights that a careful selection of hydrogel formulations for 3D bioprinting is of utmost importance. A hydrogel with a relatively high weight-to-volume ratio will form a macrostructure that can be readily stacked and shaped according to a three-dimensional model. However, the relative density of the hydrogel network will result in reduced diffusion of small molecules through the hydrogel. Conversely, a comparatively soft hydrogel will possess a less dense hydrogel network structure, resulting in enhanced diffusion of small molecules. However, this also renders it more challenging to stack in multiple layers and maintain its intended architecture. A hydrogel with a relatively open hydrogel network should therefore lead to effective diffusion of insulin and nutrients from and to hydrogel-encapsulated islets.

Here we report on a screening method for selecting promising hydrogel formulations for 3D bioprinting and the *in vitro* evaluation of the function of 3D printed insulin-secreting cells. Fluorescent recovery after photobleaching (FRAP) was utilised to gain insight into the diffusion properties of insulin-sized moieties through various alginate-based hydrogels. Rheological measurements were performed to identify the mesh size of different hydrogel formulations. [17,18]. The FRAP and rheology experiments were designed to identify a hydrogel formulation that would facilitate the diffusion of all dextrans and possess a mesh size capable of accommodating all insulin molecules. In the event of multiple formulations meeting these criteria, the one with the fastest diffusion will be selected. The 1.5 % ultrapure alginate met these criteria and was selected for further *in vitro* experimentation. INS1E cells, rat islets and human islets were subsequently 3D printed and assessed for cell viability and functionality. We have shown that careful selection of a hydrogel formulation allows for 3D bioprinted of pancreatic islets that can respond to physiological levels of glucose.

## 2 Materials and methods

### 2.1 Cell culture

INS1E β-cell line (AddexBio Technology), derived from rat insulinoma, where cultured in RPMI (Roswell Park Memorial Institute) 1640 medium (Sigma Aldrich) supplemented with 10 % fetal bovine serum (FBS, Sigma), 23.8 mM sodium bicarbonate, 10 mM (4-(2-hydroxyethyl)-1-piperazineethanesulfonic acid)) (HEPES), 1 mM sodium pyruvate, 5 mM glucose and 50 mM beta-mercaptoethanol (all Thermo Fisher Scientific).

Animal experiments were approved by the Dutch central authority for animal experiments advised by Maastricht University's animal ethics committee, application number AVD1070020186965, according to the Dutch law on animal experimentation. Rat pancreata were harvested from six ≥10-week-old male Lewis rats, perfused with 0.25 mg/mL liberase and kept on ice until digestion at 37 °C for 16 min. The digestion was stopped with quench solution (HBSS (Hanks' balanced salt solution) supplemented with 10 mM HEPES, 1 % Penicillin-streptomycin (P/S, Thermo Fisher Scientific), 2.5 mM CaCl_2_ · 2H_2_O, 4.2 mM NaHCO_3_, 1 mM MgCl2 · 6H_2_O and 10 % FBS (all Sigma)). The tissue was homogenised and filtered. Islets were purified with a ficoll gradient (Sigma-Aldrich) and centrifuged for 22 min at 1360 G at 10 °C without brakes. Islets were washed three times with quench solution and washed once with complete medium (RPMI 1640 medium supplemented with 1 % P/S, 10 % FBS, 23.8 mM sodium bicarbonate, 10 mM HEPES, 1 mM sodium pyruvate and 5 mM glucose. Islets were handpicked immediately after isolation and again the next day. Islet purity was determined with dithizone staining (Sigma-Aldrich). Roughly, 600 pancreatic islets were isolated for each rat, which translated to a total of 6860 IEQ.

Human islets (5000 IEQ) were obtained from Prodo Laboratories Inc (Irvine, CA, USA). Islets displayed a purity of 90 % and viability of 95 % before shipment. Islets were cultured in Connaught medical research laboratories (CMRL)-1066 medium (Gibco) supplemented with 0.1 % Ciprofloxacin (Sigma-Aldrich), 10 % FBS, 1 % Penicillin-streptomycin and 1 mM HEPES. Islets were allowed to rest for 1 day before seeding/printing. All primary islets were cultured in a non-treated 60 mm petri dish with 10 mL medium at 37 °C and 5 % CO_2_ with medium refreshes every other day.

### 2.2 Hydrogel preparation

Hydrogels were prepared by dissolving either sodium alginate (S-Alg) (Sigma-Aldrich) or alginate (UP-Alg, Pronova UltraPure medium viscosity grade) in different concentrations (1.5, 2.0 and 2.5 % w/v) in phosphate-buffered saline (PBS, Sigma) without MgCl_2_ and CaCl_2_ (Table 1). The powder was dissolved by stirring overnight at 35 °C. An additional condition was prepared with the addition of 5 % w/v gelatin (Sigma-Aldrich) to a 4 % w/v alginate solution and used as negative control formulation (S-Alg-G) for the hydrogel screening. This formulation was selected due to the known suboptimal insulin diffusion as previously reported by our group [19].

**Table 1:** Overview of hydrogels formulations.

**Hydrogel**	**Acronym**	**Molecular** **Weight (Mw)**	**Formulations tested**	**Experiments**
Sodium alginate	S-Alg	155 kDa	1.5 %, 2.0 %, 2.5 %	FRAP
Sodium alginate - gelatin	S-Alg-G	N/A	4 %S-Alg-5 %G	FRAP, rheology
UltraPure MVG alginate	UP-Alg	312 kDa	1.5 %, 2.0 %, 2.5 %	FRAP (all concentrations), rheology (all concentrations), cell experiments (1.5 %)

### 2.3 Bioink preparation

For bioinks the 1.5 % w/v UP-Alg formulation was selected. Three different bioinks were prepared as followed. INS1E cells were centrifuged (0.3 rcf for 5 min), medium was removed, and gently resuspended at a density of 5 million cells/mL in 1.5 % w/v UP-Alg to form the bioink for bioprinting, yielding 1.25 million cells per construct. Primary islets were incubated for 15 min at room temperature to settle the islets at the bottom of a 15 mL tube. Medium was subsequently removed, and islets were gently mixed with the 1.5 % UP-Alg hydrogel to form a second bioink. The preparation of primary islets bioink was performed with a density of 3000 IEQ/mL, yielding 300 IEQ per construct.

### 2.4 Bioprinting of hydrogels

An Ultimaker 2+ with a Discov3ry paste extruder extension kit (Structur3D printing) was used for bioprinting. The printer was cleaned with 70 % ethanol prior to all experiments. Bioinks or biomaterial inks were loaded into a 10 mL syringe under sterile conditions. A nozzle with an inner diameter of 420 μm and a x/y-speed of 1.5 mm/s was used to print the gels. The full settings used for printing all gels can be found in Supplementary Table 1. The biomaterial inks were used to prepare discs with a diameter of 28 mm of S-Alg-G, 1.5, 2, or 2.5 % w/v S-Alg and 1.5 % w/v UP-Alg were printed for fluorescent recovery after photo bleaching (FRAP). Either Ø10 mm discs or 4 × 4 grids (outer dimensions of 22 mm × 22 mm and interspacing of 5 mm) were printed with the bioinks previously mentioned for cell experiments (Supplementary Fig. 1). Small discs were printed for primary cell experiments due to limit cell numbers and reduced printing time. The printed constructs were crosslinked for 1 min in crosslinker solution (20 mM BaCl_2_, 119 mM NaCl and 10 mM MOPS (3-(N-morpholino) propanesulfonic acid) (Sigma-Aldrich). Afterwards, the constructs containing cells were washed with 9 % NaCl, PBS containing MgCl_2_ (0.1 g/L) and CaCl_2_ (0.133 g/L (all Sigma-Aldrich) and kept in the cell culture incubator at 37 °C and 5 % CO_2_ until further use.

### 2.5 Fluorescent recovery after photobleaching (FRAP)

The 28 mm diameter hydrogel discs were incubated in 0.1 mg/mL Fluorescein (FITC)-labeled dextrans with different molecular weights (either 3–5 kDa, 10 kDa, 20 kDa or 70 kDa, Sigma-Aldrich) dissolved overnight in PBS with 0.1 g/L MgCl_2_ and 0.133 g/L CaCl_2_). FRAP imaging was performed on 5 different spots in the same hydrogel with a Leica TCS SP8 STED confocal microscope with an Ø60 μm bleaching area, bleaching time of 21.2 s and imaged at a frame rate of 0.223 s for 120 s. Fluorescence recovery curves were obtained through image analysis with the open-source software FIJI (https://fiji.sc/). The time required for a bleach spot to reach half of the recovery curve (τ_1/2_) was determined through FRAPbot (http://frapbot.kohze.com/). Apparent diffusion constants (D) were determined based on the theory of Soumpasis et al.*,* [20,21] utilizing Equation (1), with r the radius of the bleach laser.

(Equation 1) D=0.224r2τ12

### 2.6 Rheology

The classical theory of rubber elasticity relates the storage modulus (G′) to the mesh size (Equation (2)), where R is the gas constant, T is the absolute temperature and N_av_ is Avogadro's number. This allows estimation of a hydrogels mesh size based on its storage modulus [22,23]. Hydrogel mesh size (D_mesh_) represents the diameter of a hard sphere that fills the void in between hydrogel network strands.

(Equation 2) $$D_{mesh}={\left(\frac{6\;RT}{\pi N_{av}G^{\prime }}\right)}^{\frac{1}{3}}$$

Equation (2) is based on the “equivalent network model” as proposed by Schurz et al. and the affine network model proposed by Rubinstein et al. [24,25]. These models cannot comprehend the complexity of actual hydrogel networks, and instead make use of an idealized infinite network. The main assumption for the affine network model is that of affine deformation: the relative deformation of each network strand is the same as the macroscopic relative deformation imposed on the whole network. Within this model, stress and deformation are related due to the shear modulus G’ (Equation (3)), where the number of network strands per unit volume (number density of strands) is $v=\frac{n}{V}$, ρ is the network density (mass per unit volume), M_s_ is the number-average molar mass of a network strand and k the Boltzmann constant.

(Equation 3) $$G^{\prime }=\frac{n\;kT}{V}=vkT=\frac{\rho RT}{M_{s}}$$

The Boltzmann constant can be rewritten into $=\frac{R}{N_{av}}$ , see Equation (4).

(Equation 4) $$G^{\prime }=v\frac{R}{N_{av}}T$$

Equation (4) can be then rewritten to make v the subject of the equation (Equation (5)).

(Equation 5) $$v=\frac{n}{V}\;\frac{N_{av}\;G^{\prime }}{RT}$$

Given that a hydrogel network is represented by a molecule junction diameter (D) and the average mesh width (M) (Fig. 2A), and the assumption that D = M, the free volume in between the network is represented by $V=\frac{\pi D_{mesh}^{3}}{6}$ [26]. When evaluating a single network strand (n = 1), $\frac{n}{V}$ can be rewritten into $\frac{6}{\pi r_{mesh}^{3}}$ , resulting in (Equation (6)), which results in Equation (2) when D_mesh_ is made the subject of the equation.

(Equation 6) $$\frac{6}{\pi D_{mesh}^{3}}=\frac{N_{av}\;G^{\prime }}{RT}$$

Rheological experiments were performed with a Discovery HR-2 hybrid rheometer and 8 mm flat plate geometry (both TA instruments). A total of 138 μL hydrogel precursor solution was casted in a 12 mm silicon mold and crosslinked in an excess of 20 mM BaCl2 for 5 min. Hydrogel discs were then loaded in the rheometer. Drying of the samples was prevented using a solvent trap filled with distilled water. Hydrogels were preconditioned with a time sweep at 1 % strain and angular frequency of 10 rad/s. Hydrogels were subjected to a frequency sweep with 1 % strain and frequencies ranging between 0.1 and 600 rad/s to determine a suitable frequency for the strain sweep. Next, hydrogels were exposed to a strain sweep with strains ranging between 0.1 and 1000 % and an angular frequency of 10 rad/s to determine the linear viscoelastic region of the hydrogels. The storage modulus measured within the linear viscoelastic region of the strain sweep was used to calculate hydrogel mesh sizes based on Equation (2).

### 2.7 Static glucose-stimulated insulin secretion (GSIS)

Single INS1E cell controls were cultivated in tissue culture-treated 6-well plates (1.25 million cells/well), while the printed constructs (1.25 million cells/construct) were placed in a cell strainer with 70-μm pores and then suspended in a 6-well plate. The cells were cultivated in 10 mL of medium and 10 mL of glucose solution was used for the GSIS.100 IEQ of free-floating islets (controls) were placed inside a Millicell cell culture insert (MERCK, 12 μm pore size, 12 mm diameter) in a 24 well plate with 500 μL of glucose solution. Constructs with 300 IEQ were also placed in in cell culture inserts and 500 μL of glucose solution was used. Kreb's buffer stock solution (25 mM HEPES, 115 mM NaCl, 24 mM NaHCO_3_, 5 mM KCl, 1 mM MgCl_2_ · 6H_2_O, 2.5 mM CaCl · 2H_2_O and 0.2 % bovine serum albumin (VWR) in sterile water) was supplemented with glucose (Sigma-Aldrich), forming either a high (16.7 mM) or low (1.67 mM) glucose solution. Cells culture medium was removed from all samples at either day 1 or day 7. Samples were then washed with low glucose solution and incubated for 2h in fresh low glucose solution at 37 °C to wash out all remaining insulin. Afterwards, samples were incubated for another 2h in fresh low glucose solution followed by 2h of incubation in high glucose solution. The samples were washed 3 × 5 min with low glucose solution and incubated for 2h in low glucose solution. The final low glucose solution of all samples was replaced for acid ethanol (1.5 % HCl (Sigma-Aldrich) in 70 % ethanol (VWR)) and incubated for 5 min to lyse the cells and release all insulin content. After each incubation step, an aliquot of glucose solution/acid ethanol was transferred to an Eppendorf tube and stored at −30 °C until the insulin ELISA was performed. ELISA kits for either human or rat insulin (Mercodia) were used to determine the insulin concentration according to manufacturer's instruction. The optical density of the samples was read at 450 nm with a spectrophotometric plate reader (CLARIOStar Plus, BMG Labtech). Single cell secretion was corrected to total insulin content and primary cell data was normalized to IEQ. Samples were diluted with Krebs buffer when needed. The stimulation index (SI) is calculated as the ratio between the high and first low.

### 2.8 Live/Dead™ viability assay

LIVE/DEAD Viability/Cytotoxicity kit for mammalian cells (ThermoFisher Scientific) was used according to manufacturer's instruction to examine the viability of printed cells. Images were taken using a Nikon Eclipse Ti inverted microscope, equipped with a Nikon DS-Ri2 camera, lumencor Sola SE II for fluorescence and a CoolLED pE100 system for diascopic white light. For fluorescent imaging, the DS-Ri2 was set to monochromatic mode. Images were taken using an excitation wavelength of 480 nm or 561 nm with FITC and TRITC emission filters respectively and a CFI Plan Fluor DL 10x objective. 5–10 images per conditions were analysed using FIJI software (https://fiji.sc/). Images chosen for figures are representative. Cell viability per image was calculated according to Equation (7) and averaged to display viability

(Equation 7) $$Viability=\left(1-\frac{{Area}_{dead}}{{Area}_{alive}}\right)100\%$$

### 2.9 Statistical analysis

Statistical analysis was performed using Graphpad Prism 8. P-values <0.05 were considered statistically significant. Direct comparison between two groups was performed by an unpaired *t*-test after assessing the assumptions of equality of variance and normality. Welch's correction was used for t-tests if the assumption of equality in variance was violated. Group comparisons were performed using one-way analysis of variance (ANOVA) with Tuckey's post hoc test after assessing the assumptions of equality of variance (Brown-Forsythe test) and normality (Shapiro-Wilk test). If the assumption of normality was not validated, the Kruskal-Wallis test in combination with Dunn's test were used. All results were reported as mean ± standard deviation (SD).

## 3 Results

During the printing process, crosslinking and subsequent experiments, the printed constructs maintained their shape (Supplementary Fig. 2).

### 3.1 Diffusion property characterisation of different alginate hydrogels with FRAP

**Fig. 1:**
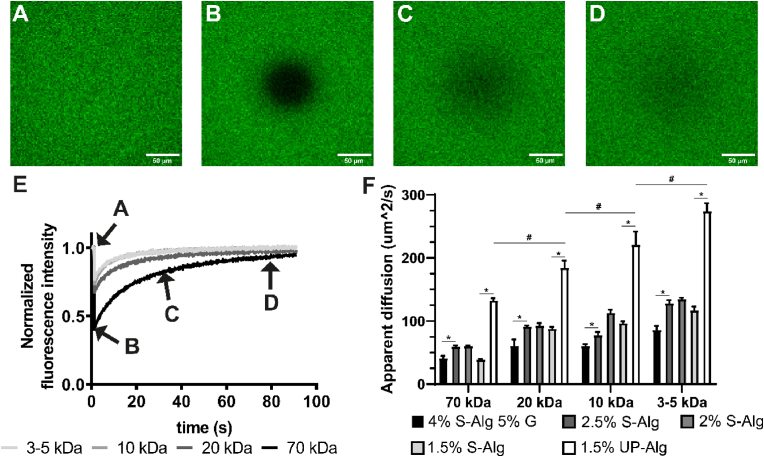
**Diffusion through a hydrogel depends on the molecular weight of FITC-labeled dextrans, the hydrogel composition and the concentration used as determined through fluorescence recovery after photobleaching (FRAP).** Representative confocal images of an ultrapure alginate (UP-Alg) hydrogel that is saturated with FITC-labeled dextran before bleaching (A), after bleaching (B), in early-recovery phase (C) and in late-recovery phase (D). The normalized fluorescence recovery curve obtained by determining fluorescence intensity of the bleached area (E), highlighting the moments at which figures A–D were taken. Obtained fluorescence recovery curves were subsequently used to calculate the apparent diffusion of FITC-labeled dextrans through the hydrogel (F), all samples were measured in 5 different locations for each condition. S-Alg = sodium alginate, UP-Alg = ultrapure alginate, G = gelatin. Data are represented as mean ± SD, ∗ indicates p < 0.05 within a dextran group, while # indicates p < 0.05 between dextran groups.

Fluorescent recovery after photobleaching imaging was used to assess the diffusion behaviour of fluorescently labeled dextran moieties with molecular sizes ranging from 3 to 70 kDa (Fig. 1). A small area of the hydrogel was imaged (Fig. 1A), after which it was bleached (Fig. 1B). Dextran moieties then diffused into the bleached area, leading to a recovery of the fluorescent signal over time (Fig. 1C and D). The fluorescent signal was quantified and normalized to the fluorescence intensity before bleaching (Fig. 1E). The apparent diffusion constant of different combinations of hydrogel configurations and dextran sizes were then evaluated (Fig. 1F). The apparent diffusion constant increased when moiety molecular weight was smaller, which was most evident in the 1.5 % w/v UP-Alg formulation, with an apparent diffusion of 132 ± 3, 184 ± 11, 221 ± 20 and 274 ± 12 μm^2^/s for 70, 20, 10 and 3–5 kDa dextrans respectively. The 4 % S-Alg - 5 % G hydrogel, previously used in 3D bioprinting of islets, showed a significant lower diffusion constant compared to the highest % w/v alginate formulation (2.5 % S-Alg), with 41 ± 4 vs 60 ± 2 μm^2^/s for 70 kDa dextran, 61 ± 10 vs 91 ± 2 μm^2^/s for 20 kDa dextran, 61 ± 3 vs 78 ± 5 μm^2^/s for 10 kDa dextran and 86 ± 6 vs 129 ± 5 μm^2^/s for 3–5 kDa). Interestingly, the hydrogel concentration did not seem to influence the apparent diffusion constants, as for instance 2.5 %, 2 % and 1.5 % S-Alg did not show differences in the diffusion of 20 kDa dextrans (91 ± 2, 93 ± 4 and 89 ± 3 respectively). Hydrogel composition, however, did influence the apparent diffusion constant, as the 1.5 % formulation of ultrapure alginate showed a significant higher diffusion constant compared to conventional sodium alginate (39 ± 1, 88 ± 3, 97 ± 3 and 117 ± 6 μm^2^/s for 70, 20, 10 and 3–5 kDa dextrans respectively). The possible destruction of the hydrogel network by the laser was evaluated by repeating FRAP measurements at the same location and comparing the obtained recovery curves (Supplementary Fig. 3). There was a 1.4 ± 0.6 % reduction in the fluorescence intensity between repeated measurements, which decreased to 0.7 ± 0.4 % after the signal was normalized to base line. The apparent diffusion of the same hydrogel determined at either one or multiple locations was statistically different (108 ± 7 μm^2^/s for same spot and 118 ± 6 μm^2^/s at different spots), a difference of 8 %.

**Fig. 2:**
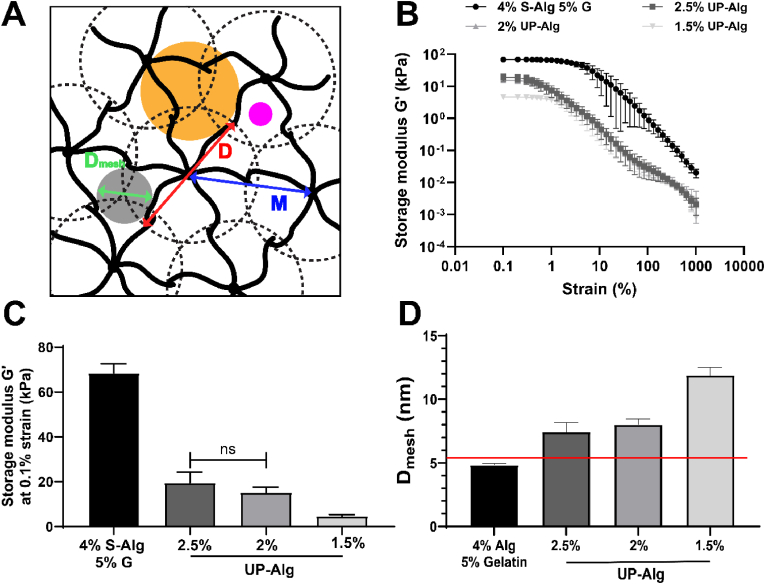
**Hydrogel mesh size is dictated by hydrogel concentration.** (A) A hydrogel network can be represented by a molecule junction diameter (D, diameter of dotted circles) and the average mesh width (M, distance in between network points). Under the assumption that D = M, the hydrogel mesh size (D_mesh_) represents the diameter of a hard sphere that fills the void in between hydrogel network strands (thick lines). The orange circle represents a molecule with limited diffusion through the hydrogel as its hydrodynamic radius ≥ D_mesh_. The magenta circle represents a molecule that is allowed to freely diffuse through the hydrogel as its hydrodynamic radius < D_mesh_. Hydrogel storage modulus during strain sweep of different alginate formulations over the whole strain range (B) and within the linear viscoelastic region at 1 % strain (C). The storage moduli displayed in figure C were subsequently used to determine the hydrogel mesh size (D) with the red line indicating the hydrodynamic radius of insulin hexamers which is 5.6 nm [27,28]. S-Alg = sodium alginate, UP-Alg = ultrapure alginate, G = gelatin. Data (N = 5) are represented as mean ± SD, ns indicates no significant statistical differences, all other groups are significantly different, p < 0.05. (For interpretation of the references to colour in this figure legend, the reader is referred to the Web version of this article.)

### 3.2 Determining the mesh size of different alginate hydrogels

The hydrogel mesh size (D_mesh_) can be envisioned as the maximum diameter of a hard sphere that fits in between the hydrogel network strands (Fig. 2A). Therefore, a strain sweep test was performed to determine G’ for different hydrogel formulations (Fig. 2B). The storage modulus in the plateau region of the strain sweep tests was quantified, with an average storage modulus of 68.4 ± 4.3, 19.5 ± 4.8, 15.2 ± 2.5 and 4.7 ± 0.7 kPa for S-Alg-G, 2.5 % UP-Alg, 2 % UP-Alg and 1.5 % UP-Alg respectively (Fig. 2C). There seems to be a trend that storage modulus decreased by decreasing hydrogel content, but there was no significant difference between 2.5 % UP-Alg and 2 % UP-Alg. The hydrogel D_mesh_ were 4.8 ± 0.1, 7.4 ± 0.7, 8.0 ± 0.4 and 11.9 ± 0.6 nm for 4 % S-Alg-5 % G, 2.5 % UP- Alg, 2 % UP-Alg and 1.5 % UP-Alg respectively (Fig. 2D). The 1.5 % UP-Alg formulation showed a significantly lower storage modulus, and therefore also a significantly higher D_mesh_ compared to all other hydrogel formulations.

### 3.3 Bioprinting INS1E single cells with 1.5 % UP alginate ink assessing viability and function

**Fig. 3:**
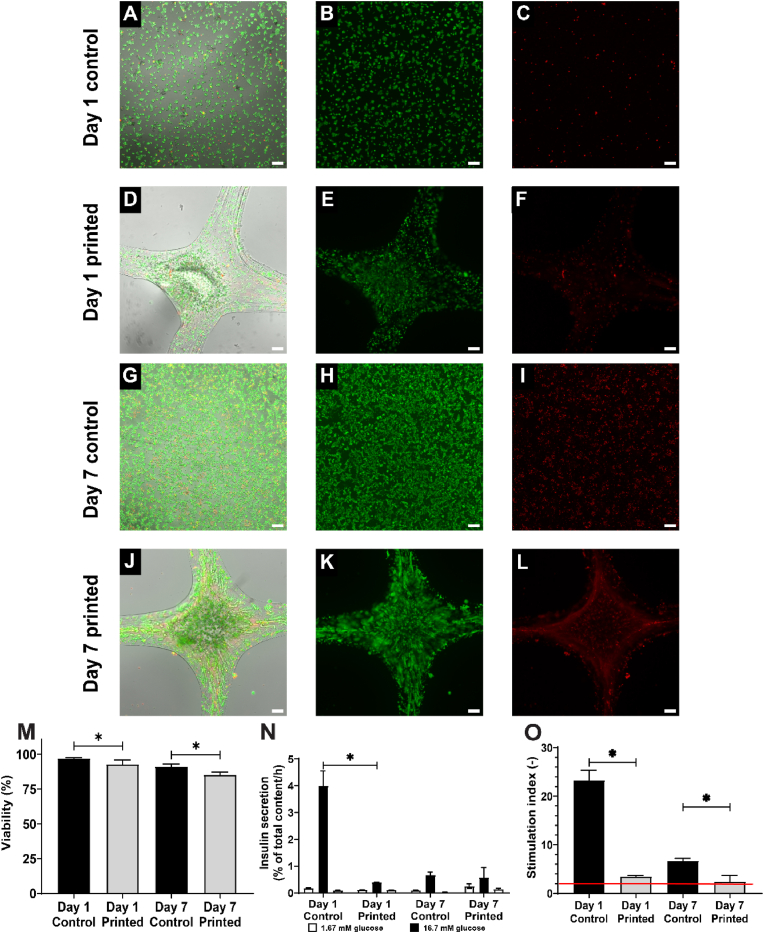
**Viability and function of INS1E cells after 3D bioprinting in 1.5 % UP alginate *in vitro*** All shown images are representative images. First column indicates the merged images, in the second column (live, green), third column (dead, red) and brightfield image. Controls cultured on regular cell culture plastic at day 1 (A–C) and day 7 (G–I), 3D bioprinted constructs containing INS1E cells at day 1 (D–F) and day 7 (J–L), and quantification of cell viability (M). Secreted insulin during a static glucose-stimulated insulin-secretion (GSIS) test by culturing alternatively in low and high glucose solutions. Decreased secretion can be seen printed condition on D1 and in both conditions in D7 (N). Stimulation indices of INS1E cells over time (O). INS1E cells displaying a stimulation index>2 (red line) were considered functional. Data (N = 3) are represented as mean ± SD, ∗ indicates p < 0.05. Scale bar 200 μm. (For interpretation of the references to colour in this figure legend, the reader is referred to the Web version of this article.)

INS1E cells were bioprinted in a 4 × 4 grid with fiber diameters of 500 μm after crosslinking. Viability of INS1E single cells were determined with live/dead staining on either day 1 or 7 of culture. The high viability of printed INS1E was evident on both D1 and D7, with values of 93 ± 3 % and 85 ± 2 %, respectively. However, these values were marginally lower compared to the controls, which showed 97 ± 1 % and 91 ± 2 %, respectively (Fig. 3A–L, M). Despite the slight decrease in viability over the 7 days of culture, high cell density was observed in both the bioprinted construct and controls (Fig. 3B–E, H, K) suggesting that the hydrogel is cell-compatible and does not hamper mass exchange of nutrients and oxygen.

**Fig. 4:**
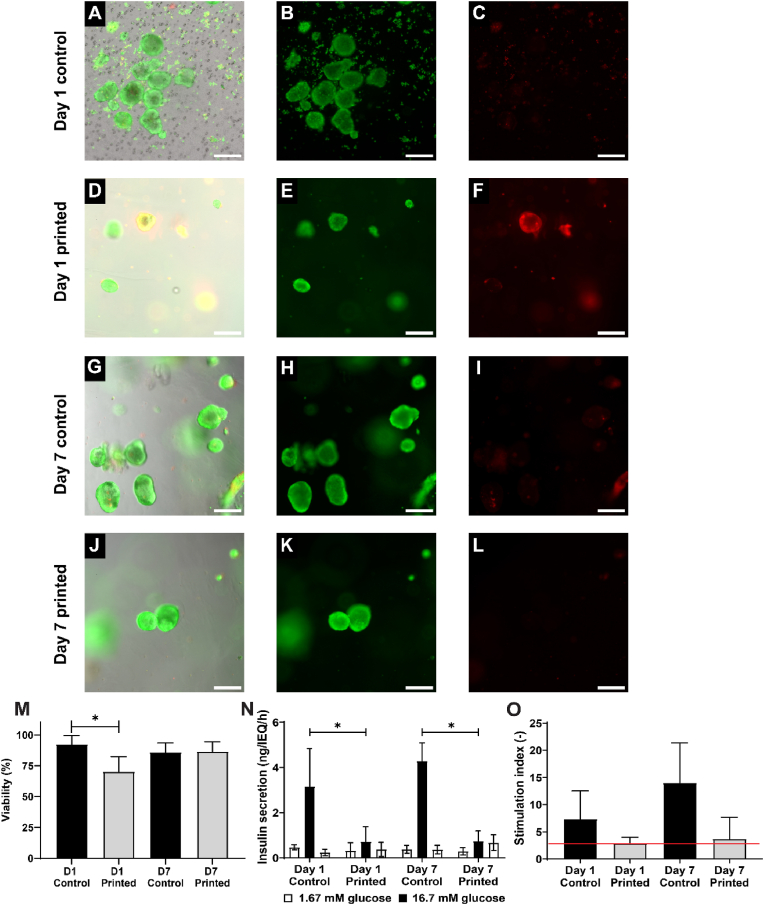
**Viability and function of rat islets after 3D bioprinting in 1.5 % UP alginate *in vitro*** All shown images are representative images. First column indicates the merged images in the second column (live, green), third column (dead, red) and brightfield image. Free-floating control islets at day 1 (A–C) and day 7 (G–I), 3D printed rat islets at day 1 (D–F) and day 7 (J–L), and quantification of cell viability (M). Secreted insulin during a static glucose-stimulated insulin-secretion (GSIS) test by culturing alternatively in low and high glucose solutions. Decreased insulin release during high glucose concentration is observed between printed and control group on D1 and D7 (N). Stimulation indices of rat islets over time (O). Rat islets displaying a stimulation index >2 (red line) were considered functional. Data (N = 5) are represented as mean ± SD, ∗ indicates p < 0.05. Scale bar 200 μm. (For interpretation of the references to colour in this figure legend, the reader is referred to the Web version of this article.)

The functionality of the INS1E cells was tested with a static GSIS. Even though the overall insulin secretion of printed constructs was less than 1 % of total insulin content, the cells displayed the expected low-high-low insulin secretion pattern (Fig. 4R). The insulin secreted during the high glucose incubation step was significantly higher for control samples compared to printed cells at day 1 (4.0 ± 0.6 % vs 0.4 ± 0.1 % of total insulin content/h), but were considered similar to printed cells at day 7 (0.7 ± 0.1 % vs 0.6 ± 0.4 % of total insulin content/h). The stimulation index of control INS1E cells was higher than printed constructs at both day 1 (23.3 ± 2.0 vs 3.5 ± 0.2) and day 7 (6.7 ± 0.5 vs 2.4 ± 1.2); nevertheless, printed INS1E cells were regarded functional as their stimulation index (SI) > 2 (Fig. 3S). Furthermore, the total insulin levels were higher at day 7 compared to day 1 for both controls and printed cells indicating high number of cells at day 7 due to cell proliferation.

### 3.4 Assessing viability and function of bioprinted rat islets

The viability and functionality of rat islets were evaluated at day 1 and 7 of culture. An average of 93 ± 7 % of islets were viable in the controls while only 70 ± 12 % of printed islets showed viable on day 1 (Fig. 4A–F, M). Printed rat islets recovered after 7 days of culture resulting in no significant difference in viability between controls (86 ± 8 %) and printed islets (86 ± 8 %) (Fig. 4G–L, M).

The functionality of the rat islets was tested with a static GSIS. The expected glucose-stimulated insulin secretion pattern was clearly observed in all conditions (Fig. 4N). The insulin secretion during the high glucose step was statistically higher in control samples compared to printed constructs at day 1 (3.1 ± 1.6 ng/IEQ/h vs 0.7 ± 0.6 ng/IEQ/h) and day 7 (4.3 ± 0.8 ng/IEQ/h vs 0.7 ± 0.5 ng/IEQ/h). The rat islets were considered functional as they displayed an SI > 2 at both day 1 (7.3 ± 5.1 for control samples, 2.9 ± 1.1 for printed constructs) and day 7 (14.0 ± 7.3 for control samples, 3.7 ± 4.0 for printed constructs) (Fig. 4O).

### 3.5 Determining viability and function of bioprinted human islets of Langerhans

**Fig. 5:**
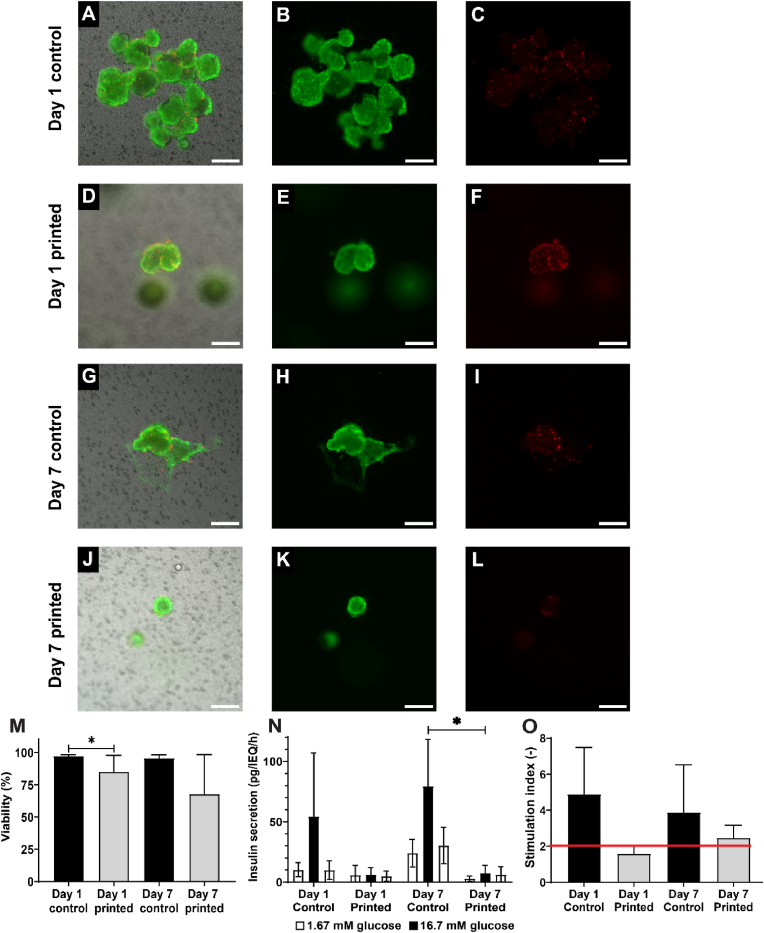
**Viability and function of human islets after 3D bioprinting in 1.5 % UP alginate *in vitro*** All shown images are representative images. First column indicates the merged images in the second column (live, green), third column (dead, red) and brightfield image. Free-floating control islets at day 1 (A–C) and day 7 (G–I), 3D printed human islets at day 1 (C–F) and day 7 (J–L), and quantification of cell viability (M). Secreted insulin during a static glucose-stimulated insulin-secretion (GSIS) test by culturing alternatively in low and high glucose solutions. Decreased insulin release insulin release is observed between control and printed condition on D1 and D7 (N). Stimulation indices of human islets over time (O). Human islets displaying a stimulation index>2 (red line) were considered functional. Data (N = 5 controls, N = 4 printed) are represented as mean ± SD, ∗ indicates p < 0.05. Scale bar 200 μm. (For interpretation of the references to colour in this figure legend, the reader is referred to the Web version of this article.)

Islets were tested for viability and functionality at either day 1 or 7 of culture. Free-floating control islets had an average viability of 97 ± 1 % while printed human islets showed an average viability of 85 ± 13 % (Fig. 5A–F, M) at day 1. After 7 days of culture, the average viability of control islets was 96 ± 3 %, while those of printed islets was 68 ± 31 % (Fig. 5G–M). Even though the average viability varies between controls and printed islets, no statistical differences were observed due to the large standard deviations in printed islets. The relatively low average viability of printed islets was a combination of islets that show high viability (around 90 %), while other islets displayed a low viability (around 40 %).

Human islet functionality was tested with a static GSIS at either day 1 or 7. The expected low – high – low insulin secretion pattern was observed at both time points (Fig. 5O). The insulin secretion of control islets during the high glucose incubation step was not statistically different to printed islets at day 1 (54.3 ± 53.0 ng/IEQ/h for control samples vs 6.2 ± 5.8 ng/IEQ/h for printed constructs) mainly due to the high standard deviation in the control samples. However, control islets showed a statistically higher insulin release during high glucose exposure compared to printed islets at day 7 (79.4 ± 38.8 ng/IEQ/h for control samples vs 7.5 ± 6.4 ng/IEQ/h for printed constructs). There was not statistical difference in SI at day 1 (4.9 ± 2.6 for control samples, 1.6 ± 0.5 for printed constructs) and day 7 (3.8 ± 2.6 for control samples, 2.5 ± 0.7 for printed constructs). The human islets of both control samples and printed constructs were considered functional at day 7 as their SI > 2.

## 4 Discussion

Even though a high cell viability was observed in previous studies [[12], [13], [14], [15], [16]], cell functionality was compromised, most likely due to impaired diffusion of insulin through the hydrogels. Careful selection of a hydrogel formulation is therefore of utmost importance to manufacture functional insulin release 3D bioprinted constructs. The aim of this study was to set-up a screening method for hydrogel formulations and select a suitable hydrogel for 3D bioprinting of pancreatic islets by showing functionality of encapsulated pancreatic islets.

### 4.1 FRAP and mesh size determination lead to the selection of 1.5 % w/v ultrapure alginate as bioink

The first step was to bioprint hydrogel discs and quantify the diffusion of fluorescently-labeled dextran moieties through the hydrogel with FRAP. A range of differently sized dextrans were used, as insulin comes in various forms. An insulin molecule is active as a monomer, but it also forms dimers and hexamers during biosynthesis and storage. Insulin monomers (size of 5.8 kDa) are formed at low concentrations (order of 1 μM), and form dimers (size of 12.4 kDa) once the concentration increases [29]. Dimers eventually combine to form hexamers (size of 36 kDa) in the presence of zinc (10 mM Zn ^2+^) and neutral pH in the secretory granule of the beta cell, where the insulin concentration is high (40 mM) [29,30]. The hexamers fall apart once secreted by beta cells, as the zinc ion concentration is relatively low in plasma, initially still forming dimers but further fragment towards monomers as the insulin gets more diluted [30]. To mimic the native situation, dextran moieties with a size of 3–5 kDa (mimicking monomers), 10 kDa (mimicking dimers), 20 kDa (size in between dimers and hexamers) and 70 kDa (mimicking larger proteins such as albumin) were used in FRAP imaging (Fig. 1). FRAP is a non-destructive technique (Supplementary Fig. 2). Even though there was a statistical difference in between samples that were measured at the same spot, or samples measured at five different locations, the difference of 8 % was minimal and is likely related to the fact that the bleached areas were not 100 % recovered after 90 s. A longer incubation time would likely have resulted in total recovery of the fluorescence curve. A free liquid control (holding a solution of the fluorescently labeled 70 kDa dextrans) was evaluated to put the apparent diffusion values of hydrogels into context. The free liquid control showed an apparent diffusion of 86750 μm^2^/s (data not shown) while the most promising hydrogel only showed an apparent diffusion of 132 μm^2^/s for this dextran size. This stresses the fact that any hydrogel will act as a diffusion barrier, slowing down the diffusion of moieties in and out of the hydrogel.

The fluorescence recovery curve, and thereby the diffusion constant, was dependent on the size of the moiety (Fig. 1E and F), as has been reported previously [31,32]. The 4 % S-Alg 5 % G hydrogel is a hydrogel formulation previously shown to maintain islet cell viability, but not functionality [14]. In the current study, the 4 % S-Alg 5 % G holds the highest hydrogel content and showed a lower diffusion constant than all other hydrogel formulations, as expected based on previous reports stressing the importance of hydrogel content [33,34]. On the same note, a difference in diffusion properties was expected between the 2.5 %, 2 % and 1.5 % S-Alg hydrogels, which however showed similar apparent diffusion values in almost all conditions. Similarly, alginate hydrogels ranging between 1.5 and 3 % alginate concentration have previously shown similar vitamin B12 (1.35 kDa) release profiles, stressing that alginate concentration marginally influences solute diffusion [35]. On the other hand, there was a large difference in diffusion constants for 1.5 % formulations of S-Alg and UP-Alg, which may be explained by their difference in molecular weight, which are 155 kDa and 312 kDa, respectively. High molecular weight alginates showed a relative higher growth factor release compared to low molecular weight alginates [9]. The relatively small molecular weight of S-Alg will likely lead to a smaller hydrogel network and therefore a lower diffusion coefficient.

However, simply choosing an alginate hydrogel with a relatively high molecular weight will not directly lead to a better bioink. Utilizing high molecular weight alginate will lead to constructs with improved physical properties, but with highly viscous, hydrogel precursor, which could generate high cell-destructive shear forces during bioprinting [5]. Diffusion kinetics of moieties through ionic hydrogels such as alginate depend on several factors of the moiety, including its hydrodynamic radius, shape, molecular weight and charge, as well as the hydrogel specific mesh size (D_mesh_). The mesh size is influenced by the concentration of hydrogel and crosslinker, hydrogel swelling ratio and environmental factors such as temperature and pH [36,37]. Mesh sizes of commonly used hydrogels have shown to range between 5 and 200 nm [10,38]. The hydrogel mesh size can be estimated based on its storage modulus. Storage moduli of alginate hydrogels have been reported ranging between 10 and 100 kPa, depending amongst others by hydrogel concentration, type of crosslinker and crosslinker concentration [39,40]. To realize sufficient diffusion of a molecule through the hydrogel, D_mesh_ should be significantly larger than the hydrodynamic radius of the molecule (Fig. 2C, magenta circle). Molecules with a hydrodynamic radius similar or larger than D_mesh_ show diminished diffusion through the hydrogel due to steric hindrance (Fig. 2C, orange circle) [17]. Several hydrodynamic radii have been reported for the different forms of insulin. Hydrodynamic radii of monomers have been reported to be 1.5 ± 0.1 nm [41] or 2.7 ± 1.4 [27], while dimers display radii of 3.0 ± 0.1 [41] or 3.8 ± 2.1 nm [27] and hexamers hold a radius of 5.6 nm [28] or 5.5 ± 1.6 nm [27]. Glucose is relatively small with a size of 0.86 nm, and is therefore not expected to experience diffusion limitations in alginate hydrogels [42]. The 4.8 nm mesh size of 4 % S-Alg-5 % G was considered prone to diffusion limitations as it was below the hydrodynamic radius of insulin hexamers, and close to hydrodynamic radii of dimers and monomers, which was confirmed by previous work of Marchioli et al. [14]. Given that the D_mesh_ of 2.5 % UP-Alg and 2 % UP-Alg are not much larger than that of 4 % S-Alg-5 % G, these hydrogel formulations were also considered prone to diffusion limitations. The 1.5 % UP-Alg formulation displayed a D_mesh_ of 11.9 nm, which is more than twice the hydrodynamic radius of insulin hexamers and was therefore considered least susceptive to diffusion limitations. The 1.5 % UP-Alg formulation was chosen to be used in *in vitro* cell work as it displayed to most promising apparent diffusion constant and D_mesh_.

### 4.2 Single cell printing as proof of principle for possible stem cell applications

INS1E cells were printed as a proof of principle for possible future applications with stem cell derived β-cells, which have shown great potential to eventually replace human islets as main cell source for cell therapies for type 1 diabetes [[43], [44], [45], [46], [47], [48], [49], [50]]. Despite allowing great perspective, there is also a risk due to the malignant potential of stem cell derived cells [51]. Incorporating the stem cells within a retrievable delivery device would therefore be optimal, with the device acting as a physical barrier preventing the unwanted roaming of cells throughout the body. It also allows for swift removal of implanted cells when side effects might occur, for instance tumor development [51]. In addition, commercial scale manufacturing of stem cell derived β-cells via aggregation protocols are currently still under development and remain challenging [52]. A potential solution would be to 3D bioprint the stem cell derived β-cells as single cells, similar to the INS1E cells printed in this work. Therefore, INS1E cells were bioprinted into a 4 × 4 grid with a density of 5 million cells/mL. The INS1E cells proliferate inside the hydrogel constructs as demonstrated by the increased cell density over the culture period and an increased total insulin content per construct at day 7 (Fig. 3B–E, H, K). The increase in cell density may also explain the decline in cell viability over the culture period observed in both control and printed samples as there is more competition for oxygen and nutrients. A general decline in viability over the course of the culture period was to be expected. The viability of the printed cells was found to be lower in comparison to the control groups on both day 1 and day 7. Nevertheless, the viability value remains high comparable to that reported in previous studies [53].

The functionality of the cells was tested with a static GSIS. The average insulin content of INS1E cells has been shown to range between 10 and 20 % of the content of native rat β-cells, with an insulin secretion around 2 % of their total insulin content/h for basal glucose levels and 12 % of their total insulin content/h for stimulated glucose levels [54,55]. Surprisingly, the overall insulin secretion during high glucose steps in controls and printed samples were considerably lower with less than 4 % of total insulin content/h and basal insulin secretion was around 0.2 % of total insulin content/h. Nevertheless, the cells displayed the expected low-high-low insulin secretion pattern (Fig. 3N). The stimulation index of printed cells was lower than controls on both day 1 and day 7, which is to be expected as the hydrogel will act as a barrier that slows down the insulin release. However, printed INS1E cells were regarded functional as their stimulation index >2 (Fig. 3O). Similar stimulation indices for INS1E cells ranging from 1.5 to 6 have been reported elsewhere [53,55,56].

### 4.3 Primary islet culture show function and support the results from FRAP and mesh size determination

A total of 300 rat or human IEQ were bioprinted in Ø10 mm discs with a density of 3000 IEQ/mL as a proof of principle for (stem cell derived) β-cell aggregates or primary islets. There was a statistical difference in islet viability between the control and the printed islets for both rat and human islets at day 1, most likely due to the fact that the printing procedure takes considerably longer than just seeding the control samples in a well plate (Fig. 4, Fig. 5A-F, M). The mild printing conditions require a slow printing speed, resulting in the fact that the final printed samples were starved from nutrients for 2 h, which likely lead to a decrease in their viability. However, there was no difference in cell viability between controls and printed samples after 7 days of culture (Fig. 4G–M), which may indicate that dead cells were washed out of the hydrogel over the culture period. Some printed human islets showed a marked decreased viability, whilst the majority of printed human islets showed a viability >90 %. This could be explained by the fact that hydrogel layers were about 500 μm thick, which could lead to a hypoxic centre layer as the maximum diffusion distance of oxygen has been described as 200 μm [57,58]. However, the current nozzle diameter was 420 μm. Decreasing the nozzle diameter would lead to thinner fibers, but would also come close to the size of islets, which are known to range between 50 and 400 μm in diameter, thereby risking the application of shear stress on the islets during printing [59].

The functionality of the primary islets was again tested with a static GSIS test. Both primary rat and human islets displayed the expected low-high-low insulin secretion pattern (Fig. 4, Fig. 5N). There seems to be less insulin secretion during the high glucose step in printed samples compared to controls. It is possible that although the mesh size of the hydrogel is large enough for insulin to pass through, other diffusion characteristics still accommodate a delayed onset of insulin secretion. This is strengthened by the fact that even after three 5-min washing steps, there seems to be residual insulin from the high glucose step left in the hydrogel, given by the relatively high insulin secretion during the second low glucose step in printed hydrogels, but not in control samples. All primary islets (controls and printed samples) were considered functional at day 7 as their stimulation indices >2, indicating that islets incorporated into the hydrogel can respond to physiological levels of glucose within a time frame of 2 h [60]. Despite the positive hydrogel diffusion and cell viability and functional outcomes observed in the current *in vitro* tests, the diffusion process in an *in vivo* environment may exhibit significant differences due to the influence of additional factors that are not possible to consider *in vitro*. These factors may include, but are not limited to, potential vascularisation, the implantation site, immune responses, and the possibility of fibrotic capsule formation. In order to assess the diffusion and function of printed constructs, an extensive *in vivo* study would be required. During an *in vivo* study, the potential for scalability issues could be identified and the possible solutions could be considered.

## 5 Conclusion

Hydrogels used for encapsulation of islets should facilitate effective diffusion of molecules from and to the encapsulated islets in order to achieve a successful extrahepatic islet transplantation alternative. For pancreatic islets, glucose needs to be able to reach the islets within the construct, which should lead to the secretion of insulin and subsequent diffusion of the insulin towards blood vessels in close proximity to the construct, effectively lowering the patients’ blood glucose levels. Any hydrogel will act as a barrier that slows down the insulin release of incorporated islets, which was clearly noticeable in GSIS assays. The difference in diffusion properties between hydrogel formulations was also clear in FRAP imaging, indicating that it is a suitable technique for the screening of hydrogel compositions for 3D bioprinting. The hydrogel with the measured best diffusion properties (1.5 % UP-Algiante) was validated with mesh size measurements and was successfully used to bioprint islets that remained viable and functional over seven days of culture. Overall, this work demonstrates that assessing hydrogel mesh size via FRAP and rheology can be used to predict the diffusion capacity of hydrogels and thereby select appropriate hydrogel compositions for 3D bioprinting applications.

## CRediT authorship contribution statement

**Carolin Hermanns:** Writing – review & editing, Writing – original draft, Methodology, Investigation, Formal analysis, Data curation. **Rick H.W. de Vries:** Writing – review & editing, Writing – original draft, Supervision, Methodology, Investigation, Formal analysis, Data curation, Conceptualization. **Timo Rademakers:** Visualization, Software, Methodology, Data curation. **Adam Stell:** Methodology. **Denise F.A. de Bont:** Methodology. **Omar Paulino da Silva Filho:** Methodology. **Marlon J. Jetten:** Methodology. **Carlos D. Mota:** Writing – review & editing. **Sami G. Mohammed:** Writing – review & editing, Methodology, Investigation, Data curation. **Vijayaganapathy Vaithilingam:** Methodology, Investigation, Data curation. **Aart A. van Apeldoorn:** Writing – review & editing, Supervision, Project administration, Funding acquisition, Conceptualization.

## Declaration of competing interest

The authors declare that they have no known competing financial interests or personal relationships that could have appeared to influence the work reported in this paper.

## Data Availability

Raw data can be made available upon request via the corresponding author and is stored at Maastricht University Datahub facilities.
