# Supplementary material for: Assessing mesh size and diffusion of alginate bioinks: A crucial factor for successful bioprinting functional pancreatic islets
Source: Mater Today Bio. 2025 Aug 5;34:102175. doi: 10.1016/j.mtbio.2025.102175 (PMC12355097; doi:10.1016/j.mtbio.2025.102175)
Supplement: Multimedia component 1 [file mmc1.docx]

**Supplementary Material**

**Assessing mesh size and diffusion of alginate bioinks: A crucial factor for successful bioprinting functional Pancreatic Islets**

Carolin Hermanns^a*^, Rick H.W. de Vries^a*^, Timo Rademakers^c^, Adam Stell^a^, Denise F.A. de Bont^a^, Omar Paulino da Silva Filho^a^, Marlon J. Jetten^a^, Carlos D. Mota^b^, Sami G. Mohammed^a^, Vijayaganapathy Vaithilingam^a^, Aart A. van Apeldoorn^a^

^a^ Department of Cell Biology – Inspired Tissue Engineering (cBITE), MERLN Institute for Technology Inspired Regenerative Medicine, Maastricht University, Maastricht, The Netherlands.

^b^ Department of Complex Tissue Regeneration (CTR), MERLN Institute for Technology Inspired Regenerative Medicine, Maastricht University, Maastricht, The Netherlands.

^c^ MERLN Institute for Technology Inspired Regenerative Medicine, Maastricht University, Maastricht, The Netherlands.

*** Authors contributed equally to this work

Correspondence to

Aart A. van Apeldoorn

Department of Cell Biology–Inspired Tissue Engineering,

MERLN Institute, Maastricht University,

PO Box 616, 6200 MD Maastricht, The Netherlands.

e-mail: [a.vanapeldoorn@maastrichtuniversity.nl](mailto:a.vanapeldoorn@maastrichtuniversity.nl)


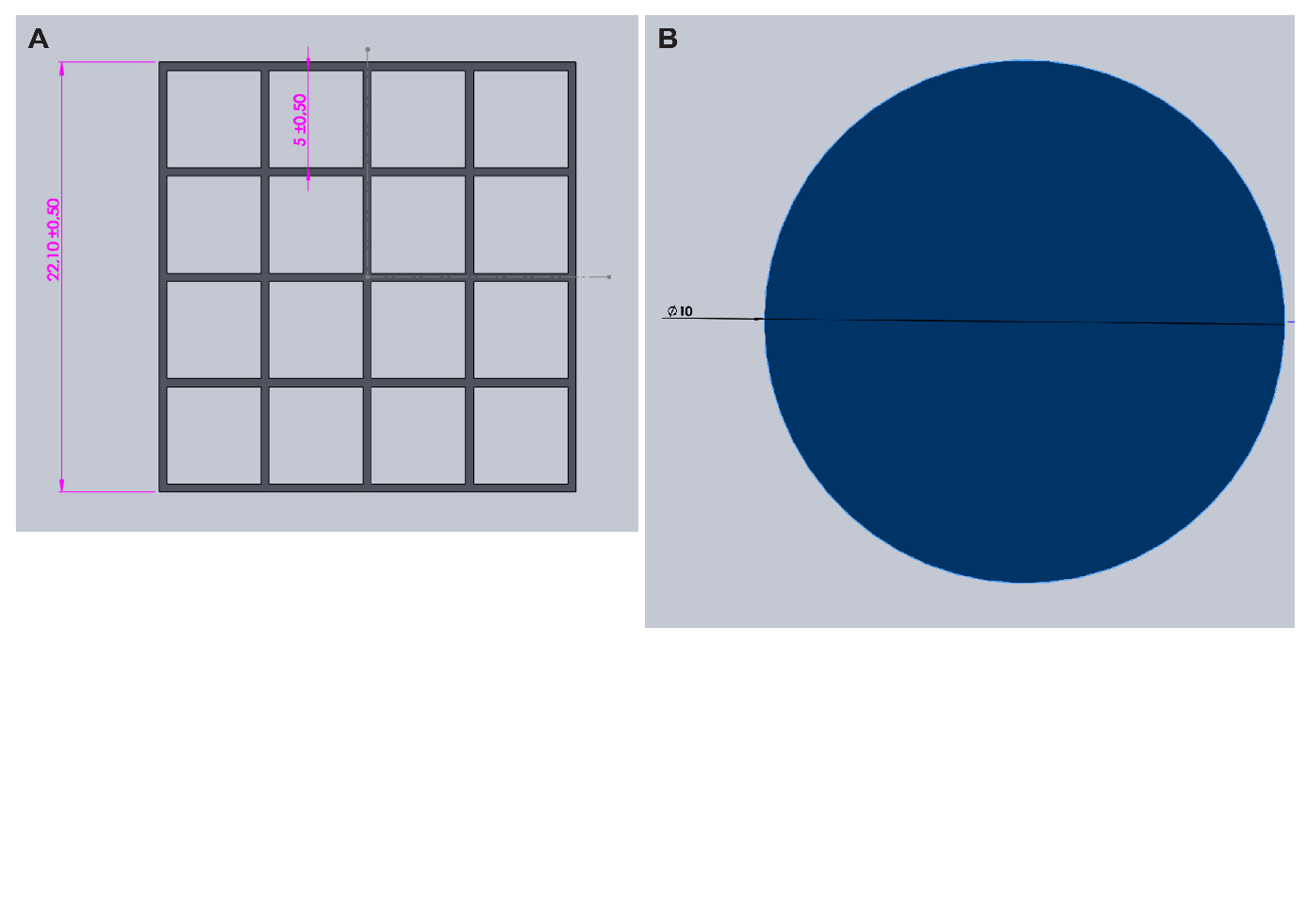


Supplementary figure 1: Schematics of the printed constructs with cells with dimensions in mm. A shows the grid and B shows the printed disc. Schematics were made in Soldiworks and slice with the cura software from UltiMaker (<https://support.ultimaker.com/s/article/1667337576727> )

**Supplementary table 1: Full printer settings in the software Cura used for printing all gels**

| Layer height | 0,41 mm |
| --- | --- |
| Initial layer height | 0,38 mm |
| Line width | 0,41 mm |
| Top/bottom line width | 0,41 mm |
| Infill line width | 0,41 mm |
| Wall thickness | 0,41 mm |
| Wall line count | 1 |
| Top/ Bottom Thickness | 0,41 mm |
| Top thickness | 0,41 mm |
| Top layers | 0 |
| Bottom Thickness | 0,41 mm |
| Bottom Layers | 999999 |
| Top/ Bottom Pattern | Lines |
| Bottom Pattern Initial Layer | Lines |
| Horizontal Expansion | 0 mm |
| Infill Density | 100% |
| Infill pattern | Cubic |
| Printing Temperature | 0,0°C |
| Build Plate Temperature | 0,0°C |
| Flow | 100% |
| Initial Layer Flow | 150% |
| Enable Retraction | unchecked |
| Print Speed | 3 mm/s |
| Travel Speed | 3 mm/s |
| Initial Layer Speed | 1.5 mm/s |
| Initial Layer Print Speed | 1.5 mm/s |
| Initial Layer Travel Speed | 1.5 mm/s |
| Enable Acceleration Control | checked |
| Print Acceleration | 3000 mm/s^2^ |
| Travel Acceleration | 200 mm/s^2^ |
| Initial Layer Acceleration | 200 mm/s^2^ |
| Enable Jerk Control | checked |
| Print Jerk | 20 mm/s |
| Travel Jerk | 30 mm/s |
| Initial Layer Jerk | 20 mm/s |
| Combing Mode | all |
| Enable Print Cooling | unchecked |
| Generate Support | unchecked |
| Build Plate Adhesion Type | None |

**
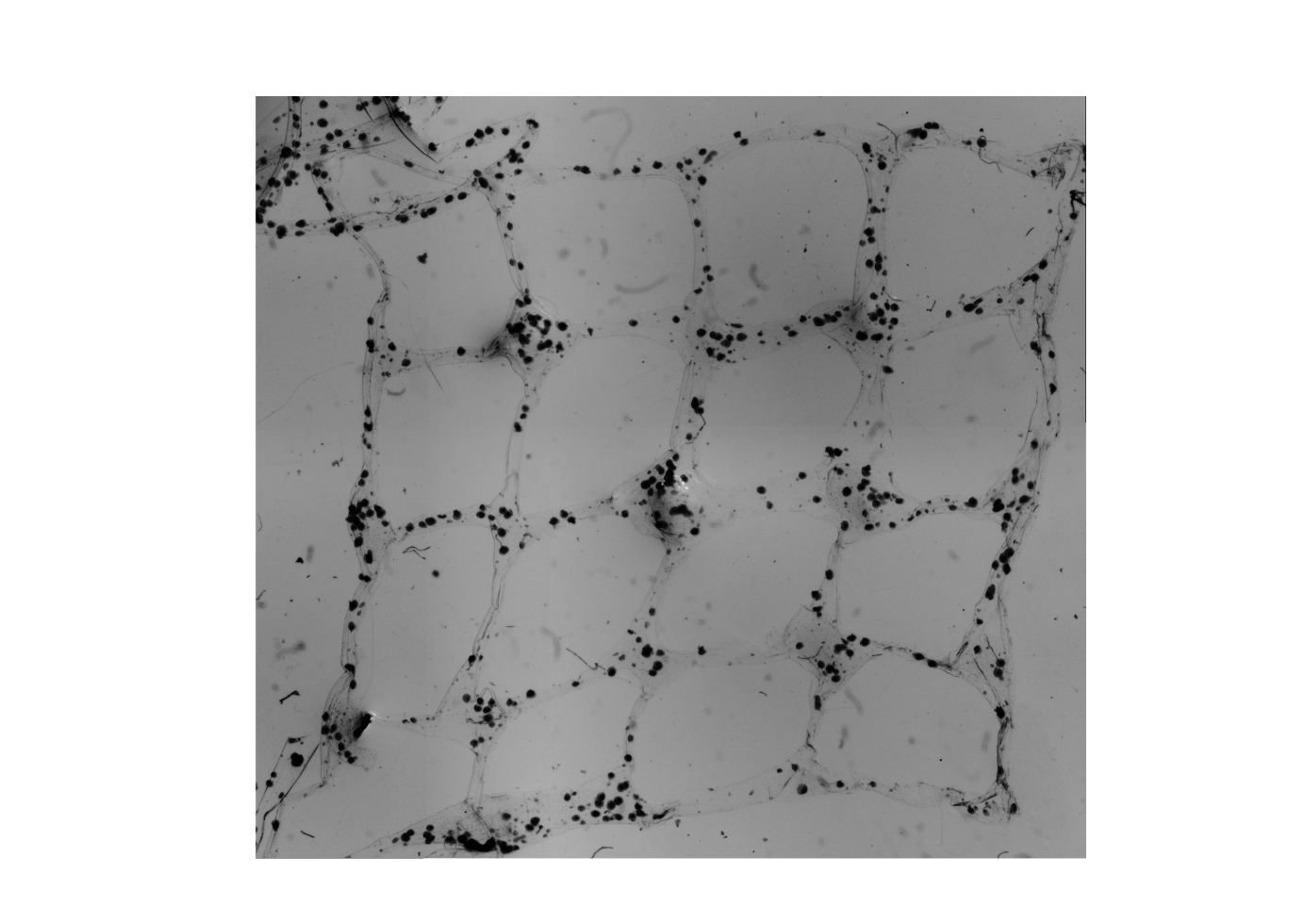
**

Supplementary figure 2: Example of a printed 4x4 grid after crosslinking and transferring from printing bed to a petri dish. The shape of the printed structure was maintained.

**
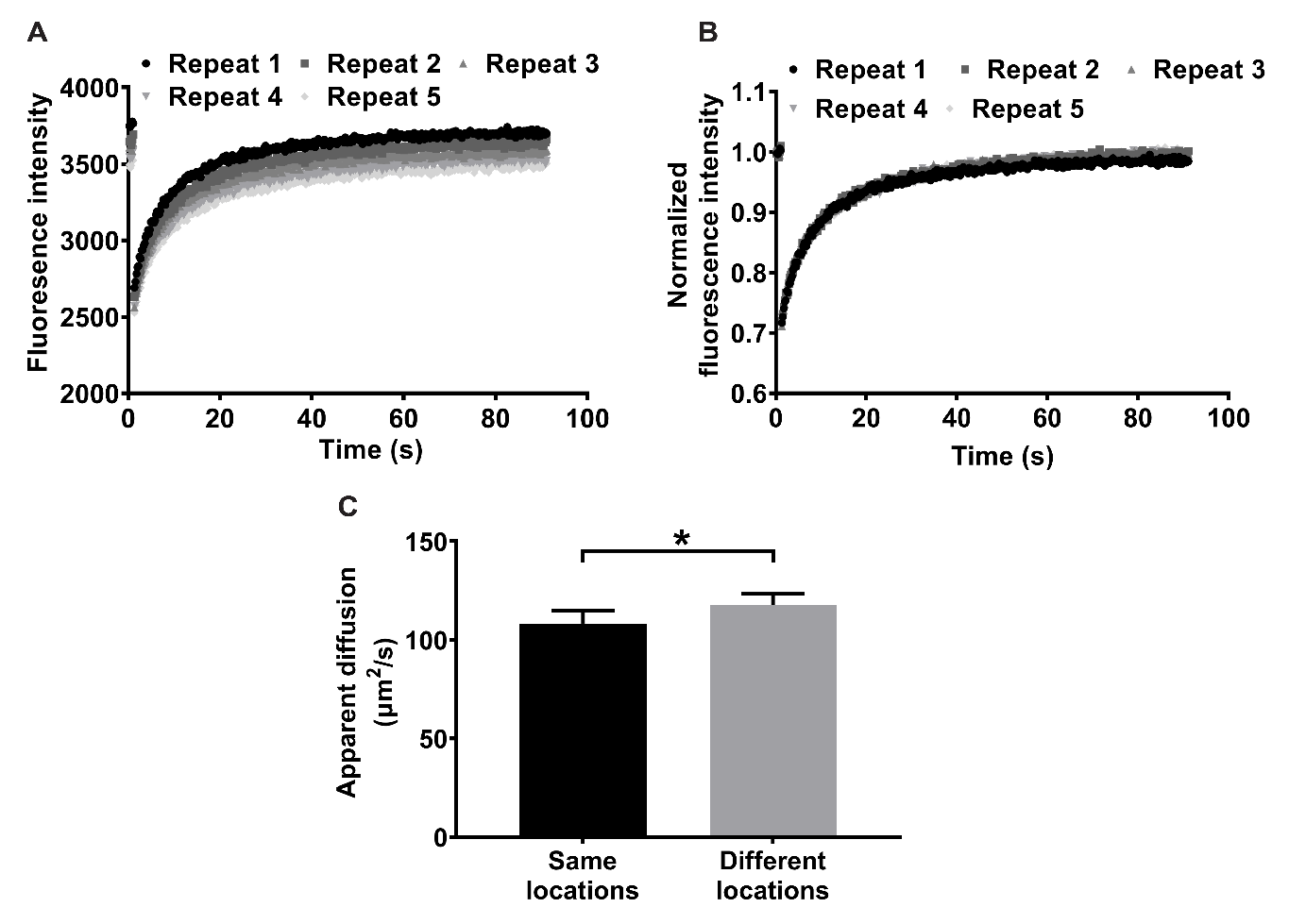
**

Supplementary figure 3: FRAP recovery curve of five repeats at the exact same location for the 1.5% w/v UP-Alg condition (A), and after normalization to base line (B). Calculated apparent diffusion of FITC-labeled dextrans through the hydrogel measured five times at the same location, or at five different locations (C).
